# Supplementary material for: Unsuppressed HIV infection impairs T cell responses to SARS-CoV-2 infection and abrogates T cell cross-recognition
Source: eLife. 2022 Jul 26;11:e78374. doi: 10.7554/eLife.78374 (PMC9355563; doi:10.7554/eLife.78374)
Supplement: Supplementary file 1. — The table contains a list of peptides spanning the receptor-binding domain (RBD) and non-RBD regions of spike with known hotspots for mutations, and a corresponding list of peptides with Beta variant lineage defining mutations. The Beta variants mutations are highlighted in red. The two sets of peptides were used for cultured expansion studies. [file elife-78374-supp1.docx]

**Supplementary file 1**

Complete list of 15mer wildtype (wt) and corresponding Beta variant S peptides sequences used for cross-recognition experiments
